# Characterization of uronate dehydrogenases catalysing the initial step in an oxidative pathway

**DOI:** 10.1111/1751-7915.12265

**Published:** 2015-04-17

**Authors:** André Pick, Jochen Schmid, Volker Sieber

**Affiliations:** Wissenschaftszentrum Straubing, Lehrstuhl für Chemie Biogener Rohstoffe, Technische Universtität MünchenSchulgasse 16, Straubing, 94315, Germany

## Abstract

Uronate dehydrogenases catalyse the oxidation of uronic acids to aldaric acids, which represent ‘top value-added chemicals’ that have the potential to substitute petroleum-derived chemicals. The identification and annotation of three uronate dehydrogenases derived from *Fulvimarina pelagi* HTCC2506, *Streptomyces viridochromogenes* DSM 40736 and *Oceanicola granulosus* DSM 15982 via sequence analysis is described. Characterization and comparison with two known uronate dehydrogenases in regard to substrate spectrum, catalytic activity and pH as well as temperature dependence was performed. The catalytic efficiency was investigated in two different buffer systems; potassium phosphate and Tris-HCl. In addition to the typical and well available substrates glucuronate and galacturonate also mannuronate as part of many structural polysaccharides were tested. The uronate dehydrogenase of *Agrobacterium tumefaciens* and *Pseudomonas syringae* showed catalytic dependency on the buffer system resulting in an increased *K_m_* especially for glucuronate in potassium phosphate compared with Tris-HCl buffer. Enzyme stability at 37°C of the different Udhs was in the order: *P. syringae* < *S. viridochromogens* < *A. tumefaciens* < *F. pelagi* < *O*. *granulosus*. All enzymes showed activity within a broad pH range from 7.0 to 9.5, only *O. granulosus* had a very narrow range around 7.0.

## Introduction

Uronate dehydrogenase (Udh, EC 1.1.1.203) catalyses the dehydrogenation of uronic acids to aldaro-lactones. When these are hydrolysed aldaric acids are formed that can be applied for example as biobased chelating agents or for the production of building block monomers for polyamides or polyesters and therefore are considered as ‘top value-added chemicals’ for a biobased economy (Werpy *et al*., 2004). This reaction represents the first step in an oxidative pathway for utilization of uronic acids and was first described in the phytopathogenic bacteria *Pseudomonas syringae* and *Agrobacterium tumefaciens* (Zajic, 1959).

Uronic acids such as glucuronate (GlcA) and galacturonate (GalA) are ubiquitously distributed sugar derivatives. They represent monomeric building blocks of structural polysaccharides such as hemicellulose and pectin (Saha, 2003). They are also present in macroalgae and microalgae, which are considered as feedstock of third generation biorefineries (Lahaye and Robic, 2007; Jung *et al*., 2012). Additionally, they are components of extracellular polymeric substances responsible for microbial biofilm formation (Sutherland, 2001). Furthermore, in mammals, GlcA represents an intermediate of vitamin C biosynthesis and therefore is used as detoxificating and eliminating agent of endobiotics and xenobiotics via glucuronidation (Mulder, 1992; Guéraud and Paris, 1998; Linster and Van Schaftingen, 2007).

Because of the widespread occurrence many microorganisms are able to use uronic acids as carbon and energy source (McRorie and Novelli, 1958; Farmer and Eagon, 1969). Several catabolic pathways exist, which guide these compounds towards the central metabolism (Ashwell *et al*., 1958; Richard and Hilditch, 2009). Examinations that dealt with the characterization of uronate dehydrogenases and the subsequent enzymatic steps involved, revealed two oxidative pathways that lead to the intermediates α-ketoglutaric acid or pyruvate and L-tartronate semialdehyde (Bateman *et al*., 1970; Chang and Feingold, 1970; Wagner and Hollmann, 1976; Richard and Hilditch, 2009). The oxidative pathway for glucuronic and galacturonic acid towards α-ketoglutaric acid is similar to an alternative C_5_-carbohydrate utilization as identified for xylose and arabinose (Watanabe *et al*., 2006a,b; Stephens *et al*., 2007). Initial characterization of uronate dehydrogenases derived from *P. syringae* and *A. tumefaciens* was performed without knowledge of the corresponding amino acid sequence of the enzymes. The genes were recently identified for the uronate dehydrogenases of *Pseudomonas putida*, *A. tumefaciens* and *P. syringae* using a complementation assay (Yoon *et al*., 2009). The identification was directly linked to a metabolic engineering approach aimed to design a synthetic pathway for the production of glucaric acid from glucose as substrate (Moon *et al*., 2009). In addition, fungal strains, which are capable to produce *meso*-galactarate by use of uronate dehydrogenase, were developed (Mojzita *et al*., 2010). Recently, the crystal structures of two uronate dehydrogenases from *A. tumefaciens* and *Chromohalobacter salexigens* DSM 3043 were elucidated (Ahn *et al*., 2011; Parkkinen *et al*., 2011). The increased interest on enzymes and pathways converting sugar and sugar derivatives reflects the need of biotechnological conversion routes of biogenic raw materials into chemicals and fuels (Grohmann *et al*., 1998; Carvalheiro *et al*., 2008). The aim of this study was to identify uronate dehydrogenases on basis of a blast search and subsequently characterize them with a focus on kinetic parameters and substrate specificity. The candidates were finally evaluated for biocatalyst-based conversion routes towards valuable chemical building blocks.

## Results

### Selection of Udhs

**Fig 1:**
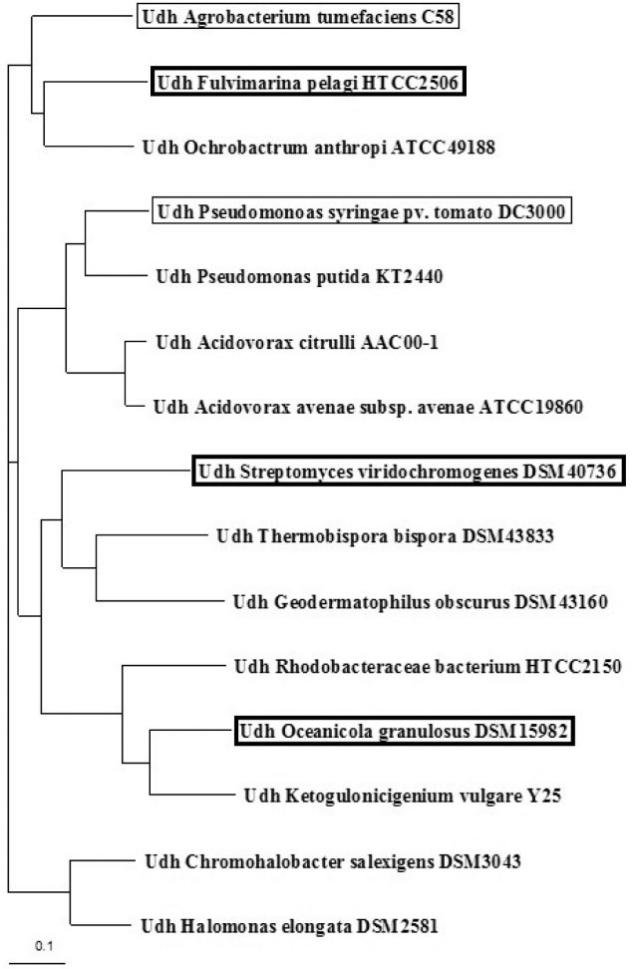
Phylogenetic tree of investigated uronate dehydrogenases and putative enzymes identified through blast. The two reference enzymes are thin framed, and the three investigated enzymes are thick-framed. The alignment was calculated with clustalw2, and for visualization the program treeview was used.

The selection of the uronate dehydrogenase candidates was performed by a blast analysis based on the amino acid sequences of the three enzymes derived from *A. tumefaciens*, *P. putida* and *P. syringae* identified by Yoon and colleagues (2009). In a first screening, nine potential candidates belonging to different species were chosen on the basis of their maximum identity to the query sequence and an alignment, and a phylogenetic tree calculation (Fig. 1) was performed. Complemented by the Udh of *C. salexigens* for which a crystal structure is available, a final selection was conducted (Ahn *et al*., 2011). The putative uronate dehydrogenase of *F. pelagi* HTCC2506 was chosen because of the close proximity to *A. tumefaciens* C58, which exhibits the highest catalytic activity (Yoon *et al*., 2009). Additionally, two further candidates, *S. viridochromogenes* DSM 40736 and *O. granulosus* DSM 15982, were selected with a lower relationship in order to extend sequence diversity.

### Heterologous expression

The codon-optimized *udh* genes of *A. tumefaciens*, *P. syringae* and *F. pelagi* as well as the wild-type genes for *udh* of *S. viridochromogenes* and *udh O. granulosus* were heterologously expressed in *E. coli* BL21 (DE3) in different variants. Untagged versions as well as His-tagged versions (C- and N-terminal) were constructed for all genes of interest. Solely the C-terminal His-Tag version of *udhSv* was not obtained, due to a stop-codon in the reverse primer. Comparing expression in Luria-Bertani media (LB) and autoinduction media revealed an increased production of soluble enzyme for all five N-terminal His-Tag variants in autoinduction media. Based on this finding expression without IPTG induction was performed for further characterization. Expression of the C-terminal His-Tag variants of UdhAt and UdhFp in autoinduction media was not possible (data not shown). All native constructs without additional His-Tag modifications could be expressed as soluble protein. Expression of N-terminal His-Tag constructs in autoinduction media still resulted in the formation of inclusion bodies. Distribution between insoluble and soluble protein varied from almost complete solubility of UdhAt to a 50:50 ratio for UdhSv and UdhOg (data not shown). The kinetic parameters of the different Udhs were determined for the purified N-terminal His-Tag variants. Eluted proteins appeared as single band on SDS polyacrylamide gels. Enzyme activities of the different Udhs were stable after storage at 8°C in desalting buffer (50 mM Tris-HCl, pH 8.0) for at least 7 days. Prolonged storage time resulted in decreased enzyme activity of all variants, especially for UdhOg. Long-term storage at −20°C (4 month) was realized without any loss of activity by use of 25% (v/v) glycerol.

### Substrate and cofactor specificity of Udhs

Purified enzymes were first tested with d-glucuronic acid and d-galacturonic acid. Both were accepted as substrates by all enzymes. According to this, kinetic parameters *k_cat_* and *K*_m_ for the two substrates of all five enzymes were determined (Table 1). Additionally, glucose and galactose were tested as possible substrates. An enzyme activity was detectable for all five enzymes, but it was decreased by a factor of thousand compared with d-glucuronic acid and d-galacturonic acid. Substitution of Nicotinamide adenine dinucleotide (NAD^+^) by Nicotinamide adenine dinucleotide phosphate (NADP^+^) led to a decrease of more than 99% of the activity for all enzymes. Kinetic constants of UdhAt (GlcA: *K_m_* 0.37 mM and *k_cat_* 190 s^−1^; GalA: *K_m_* 0.16 mM and *k_cat_* 92 s^−1^; NAD^+^: *K_m_* 0.18 mM) and UdhPs (GlcA: *K_m_* 0.28 mM and *k_cat_* 74 s^−1^; GalA: *K_m_* 0.04 mM and *k_cat_* 24 s^−1^; NAD^+^: K*_m_* 0.17 mM) were described by Yoon and colleagues (2009). In comparison with these results in our experiments, both kinetic constants, *k_cat_* as well as *K_m_*, had slightly decreased values. In order to find optimal conditions for the enzymes' application, we analysed the effect of an inorganic and organic buffer system. UdhAt and UdhPs revealed clear dependency of *K_m_* and *k_cat_* on the buffer system. This effect was most significant for UdhAt with glucuronic acid as substrate, which resulted in a fivefold increase of *K_m_* for glucuronic acid (1 mM) in KP_i_ buffer compared with Tris buffer (0.2 mM). An increase of *k_cat_* was observed for d-glucuronic acid in KP_i_ buffer. This effect was less obvious for galacturonic acid. UdhPs showed a similar behaviour for glucuronic acid as substrate concerning the *K_m_* value. For galacturonic acid, the *K_m_* was not subjected to the buffer system.

**Table 1:** Kinetic parameters determined for the different uronate dehydrogenases in dependency of two buffer systems

Strain and Substrate	KP_i_-Buffer			Tris-Buffer		
	Kinetic parameters			Kinetic parameters		
	k*_cat_* (s^−1^)	*K_m_* (mM)	k*_cat_*/*K_m_* (s^−1^ mM^−1^)	k*_cat_* (s^−1^)	*K_m_* (mM)	k*_cat_*/*K_m_* (s^−1^ mM^−1^)
*A. tumefaciens*						
Glucuronate	333.0 ± 9.0	1.0 ± 0.1	342	171.0 ± 3.0	0.20 ± 0.02	829
Galacturonate	59.0 ± 3.0	0.3 ± 0.1	200	76.0 ± 2.0	0.10 ± 0.02	646
Mannuronate	85.0 ± 7.0	19.4 ± 0.6	4	92.0 ± 1.0	8.30 ± 0.20	11
NAD^+^	144.0 ± 14.0	0.1 ± 0.0	1083			
*F. pelagi*						
Glucuronate	82.0 ± 2.0	0.30 ± 0.10	274	58.0 ± 2.0	0.20 ± 0.03	269
Galacturonate	13.0 ± 1.0	0.03 ± 0.01	406	10.0 ± 0.3	0.02 ± 0.00	430
Mannuronate	107.0 ± 1.0	9.50 ± 0.20	11	78.0 ± 2.1	6.40 ± 0.13	12
NAD^+^	113.0 ± 5.0	0.20 ± 0.02	785			
*O. granulosus*						
Glucuronate	6.0 ± 0.2	59.0 ± 6.0	0.11	7.0 ± 0.1	64.00 ± 3.00	0.10
Galacturonate	3.0 ± 0.1	64.0 ± 4.0	0.04	4.0 ± 0.1	55.00 ± 3.00	0.07
Mannuronate	4.0 ± 0.1	142.0 ± 5.0	0.03	3.4 ± 0.1	112.00 ± 4.00	0.03
NAD^+^	7.0 ± 0.4	0.4 ± 0.1				
*S. viridochromogenes*						
Glucuronate	75.0 ± 2.3	0.3 ± 0.1	257.0	53.0 ± 0.6	0.30 ± 0.04	201.0
Galacturonate	17.0 ± 0.5	0.2 ± 0.0	88.0	23.0 ± 0.3	0.20 ± 0.02	98.0
Mannuronate	9.0 ± 0.1	13.6 ± 0.1	0.7	8.0 ± 0.3	12.30 ± 0.60	0.7
NAD^+^	62.0 ± 1.6	0.1 ± 0.0	638.0			
*P. syringae*						
Glucuronate	108.0 ± 4.0	0.50 ± 0.02	210.0	63.0 ± 1.1	0.14 ± 0.04	465.0
Galacturonate	29.0 ± 1.0	0.04 ± 0.01	794.0	21.0 ± 0.3	0.03 ± 0.01	628.0
Mannuronate	7.0 ± 1.0	22.05 ± 1.20	0.3	6.4 ± 0.1	7.30 ± 0.40	0.9
NAD^+^	215.0 ± 12.0	0.20 ± 0.03	1262.0			

A higher affinity towards GalA as reported by Yoon and colleagues (2009) was not observed for any of the uronate dehydrogenases examined. For the reference enzymes (UdhAt and UdhPs), the preference of galacturonic acid was confirmed. Additionally UdhFp showed a lower *K_m_* for GalA. UdhSv and UdhOg showed almost no difference in the *K_m_* for both substrates. The largest *K_m_* for both substrates in combination with the lowest *k_cat_* was found for UdhOg. The determination of the *K_m_* for NAD^+^ revealed a similar cofactor affinity with one exception. The Michaelis–Menten constant for NAD^+^ of UdhOg (0.4 mM) was at least double that of the remaining enzymes (0.1–0.17 mM). Comparison of first-order rate constants (*k_cat_*/*K_m_*) allowed the division into two groups; UdhAt, UdhSv and UdhOg shared the highest catalytic activity with GluA, whereas UdhFp and UdhPs preferred GalA as substrate.

Based on the observation that UdhOg has only very low activity on both substrates, we assumed that the natural substrates of this enzyme could be different uronic acids. *Oceanicola granulosus* is of marine origin. We therefore speculated that this enzyme could be more proficient in oxidizing mannuronic acid, which is part of alginate, a major constituent of brown seaweeds. Mannuronic acid is not readily available from commercial resources. We therefore prepared this compound from alginic acid ourselves. Determination of kinetic parameters for d-mannuronic acid as the third substrate for all enzymes, however, revealed no changed substrate preference. Again, UdhAt and UdhPs showed a different behaviour concerning the *K_m_* depending on the used buffer system. UdhFp had the best catalytic activity in both buffer systems based on the five tested enzymes. The differences concerning the *K_m_* depending on the substrate is the lowest for UdhOg and only increased by a factor of 2 compared with GlcA. The remaining four enzymes show at least an increase by a factor of 20 for mannuronate as substrate compared with GlcA.

### Influence of temperature and pH on enzyme activity

**Fig 2:**
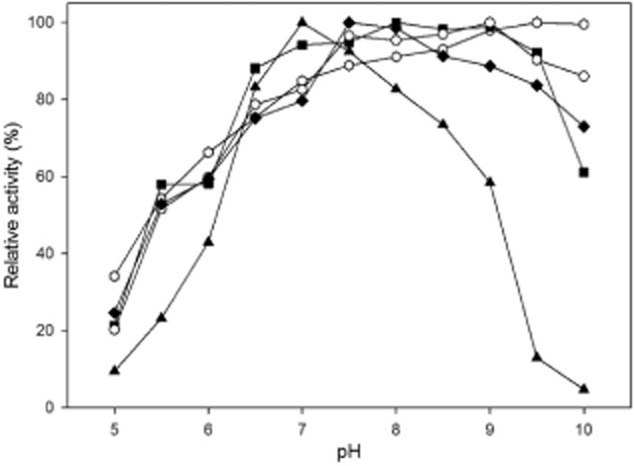
Effect of pH on activities of Udhs from *A. tumefaciens*, *F. pelagi*, *O. granulosus*, *S. viridochromogenes* and *P. syringae* presented as relative activities. Hexagon, *F. pelagi* UDH; Squares, *A. tumefaciens* UDH; Triangles, *O. granulosus* UDH; Circles, *S. viridochromogenes* UDH; Diamonds, *P. syringae* UDH.

The parameters pH and temperature were investigated with respect to their influence on the enzyme activity. pH-dependent enzyme activity (Fig. 2) was measured in steps of 0.5 in the range from 5 to 10. UdhFp was most active under alkaline conditions with highest enzyme activity at pH 9.5 to pH 10.0. At a pH higher than 10.0, the enzyme activity of UdhFp also decreased rapidly (data not shown). Interestingly, in comparison with the other enzymes tested, UdhFP also had the highest activity under acidic conditions. At pH 5, around 30% of the maximal activity remained. UdhAt demonstrated optimal enzyme activity at pH 8 with a flat plateau showing significant decrease below pH 6.5 and above pH 9.5. A similar behaviour was observed for UdhPs and UdhSv with a slight shift of maximum activity towards alkaline conditions. UdhOg showed a completely different behaviour. Optimal enzyme activity was reached at pH 7.0, and only in a small range between pH 6.5 and 7.5, the enzyme exhibited more than 90% of its activity. Above pH 7, a constant decrease in enzyme activity with a significant drop at pH 9 was observed.

**Fig 3:**
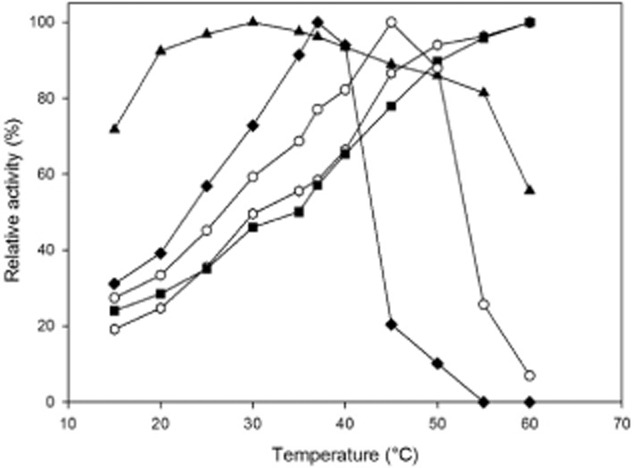
Effect of temperature on activities of Udhs from *A. tumefaciens*, *F. pelagi*, *O. granulosus*, *S. viridochromogenes* and *P. syringae* presented as relative activities., Hexagon, *F. pelagi* UDH; Squares, *A. tumefaciens* UDH; Triangles, *O. granulosus* UDH; Circles, *S. viridochromogenes* UDH; Diamonds, *P. syringae* UDH.

**Fig 4:**
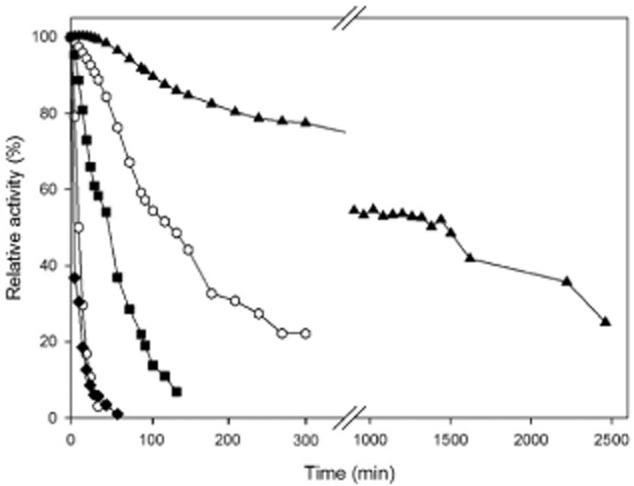
Effect of incubation at 37°C on activities of Udhs from *A. tumefaciens*, *F. pelagi*, *O. granulosus*, *S. viridochromogenes* and *P. syringae* presented as relative activities. Hexagon, *F. pelagi* UDH; Squares, *A. tumefaciens* UDH; Triangles, *O. granulosus* UDH; Circles, *S. viridochromogenes* UDH; Diamonds, *P. syringae* UDH.

The influence of temperature on enzyme activity was investigated between 15°C to 60°C (Fig. 3). UdhFp and UdhAt showed a constant increase in activity with rising temperature up to 60°C, no maximum was reached within these experiments. In the range of 15–45°C, UdhSv also showed a constant increase, however with a maximum at 45°C above which a rapid decrease in activity occurred. A similar temperature profile was found for UdhPs with the exception of this enzyme being more labile towards temperature changes and the maximum activity being reached at 37°C already. The activity drops rapidly at higher temperatures, and at 55°C, a complete loss of enzyme activity occurred. In contrast, UdhOg revealed a completely different profile. Maximal enzyme activity was reached at 30°C with a plateau of at least 90% activity in the range of 20–40°C. Starting at 30°C, activity slowly decreased until 55°C and dropped rapidly at 60°C to a residual activity of 50%. Additionally, enzyme stability at 37°C was investigated (Fig. 4). UdhPs and UdhSv accordingly showed the lowest stability among the five candidates. After 30 min, the activity of both enzymes had almost completely disappeared. The loss of initial activity was faster for UdhPs (50% activity after 2 min). For UdhSv, it took over 10 min until the activity fell below 50%. Uronate dehydrogenase of *A. tumefaciens* exhibited a more than three times higher half-life (ca. 50 min) compared with UdhSv and UdhPs. *Fulvimarina pelagi* uronate dehydrogenase displayed a higher stability with a t½ of 120 min. The highest thermal stability, which was not affected through substrate concentration, was shown by UdhOg with a half-life of more than 20 h.

### Comparison of Udhs

**Fig 5:**
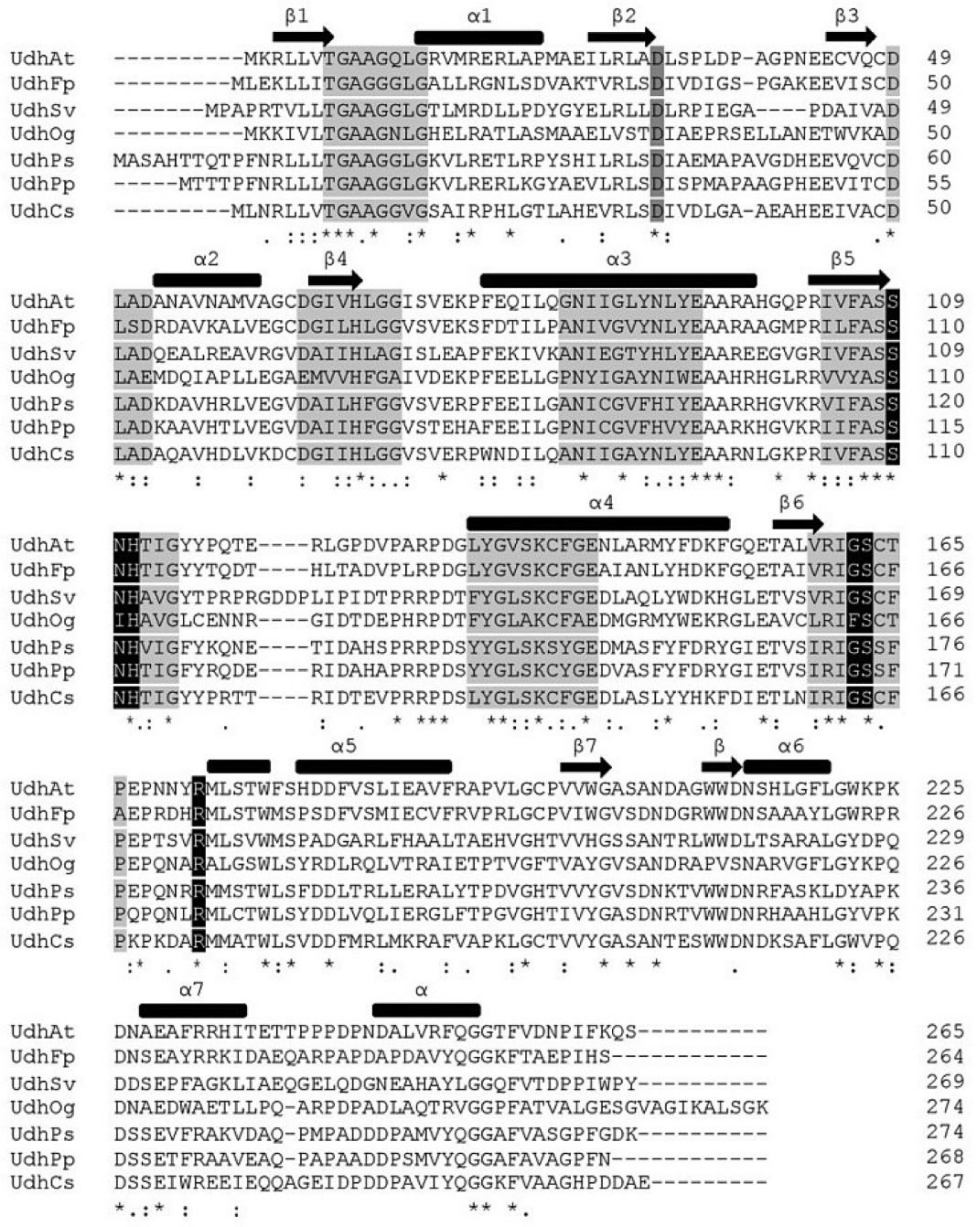
clustal omega alignment of known uronate dehydrogenases (Sievers *et al*., 2011). The secondary structures are shown above with thick bars for α-helices and arrows for β-sheets. Coloured residues represent conserved sequence motifs within the SDR family and also residues involved in the specific substrate and cofactor recognition.

All chosen uronate dehydrogenases were compared via clustal omega alignment (Fig. 5). They are all belonging to the short-chain dehydrogenase/reductase (SDR) family (Ahn *et al*., 2011; Parkkinen *et al*., 2011) and, based on the conserved motifs identified by Jörnvall and Persson (in Persson *et al*., 2003), are part of ‘Intermediate’ family.

**Fig 6:**
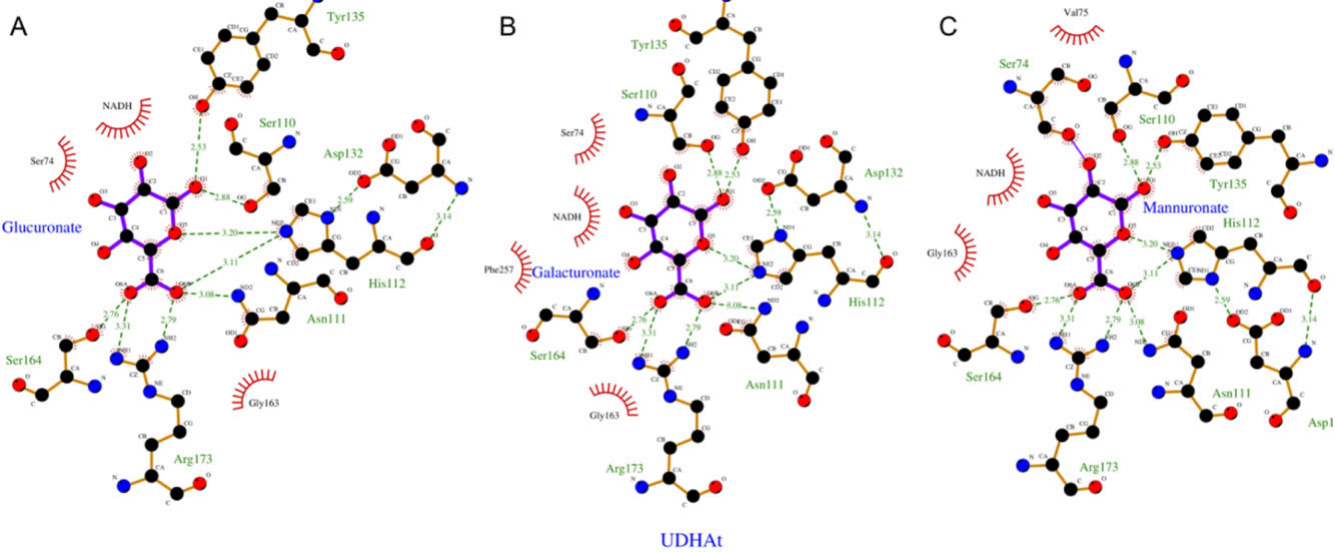
2D-Plots for the substrate binding of UdhAt on basis of PDB entry 3RFV generated by ligplot+ (Laskowski and Swindells, 2011). The initial substrate galacturonate (B) was converted manually towards glucuronate (A) and mannuronate (C) and examined for differences in the substrate binding.

Computational models for UdhFp, UdhPp, UdhPs, UdhSv and UdhOg were created by the use of Phyre2 on the basis of the available crystal structures for UdhAt and UdhCs (Kelley and Sternberg, 2009). The following order based on highest identity to the available structures was observed: UdhFp (59% UdhAt) > UdhPp (55% UdhCs) = UdhPs (55% UdhCs) > UdhSv (47% UdhCs) > UdhOg (41% UdhAt). Comparison of the deduced models and the sequence alignment was performed to identify residues that are potentially responsible for the substrate binding. The conserved sequence motifs of the β5- and β6-sheet harbour most of the residues for substrate identification/recognition and stabilization (Ahn *et al*., 2012). Within the β5-sheet (AS 111 in UdhAt) of UdhOg, an isoleucine was observed instead of an asparagine as present at the corresponding position for all other enzymes examined. Additionally, the residues Ser110, His112 of UdhAt interact with the substrate apparent from Fig. 6. In the β6-sheet, a conserved glycine (Gly163 UdhAt) residue is changed into phenylalanine. Supported by the 2D-Plots generated with LigPlot + differences in the interaction between the three substrates within the active centre shown in Fig. 6 could be identified (Laskowski and Swindells, 2011). Exemplarily, UdhAt was used to describe the diverging interactions. GluA and GalA differ in the stereochemistry of the C4 hydroxy group, which results in an additional hydrophobic contact through Phe257. This could possibly lead to a better affinity of GalA within the active centre. The observed difference for mannuronate (ManA) as substrate results from a repelling interaction between Ser74 and the C2 hydroxy group.

## Discussion

The focus of this work was the identification and characterization of uronate dehydrogenases as potential biocatalysts for biomass conversion. Three already characterized uronate dehydrogenases by Yoon and colleagues (2009) were used as references for blast analysis. Based on high sequence homology, three further uronate dehydrogenases were identified. For a straight comparison, also the previously published UdhAt and UdhPs were characterized. All five enzymes share the same substrate spectra, being specific for uronic acids. Catalytic activities for all three substrates revealed a significant difference among UdhAt, UdhSv and UdhOg, which showed a better performance with GlcA compared with UdhFp and UdhPs, which favour GalA. These finding is in contrast to the results of Yoon and colleagues (2009) who described a better performance with galacturonic acid as substrate for UdhAt and UdhPs. This variation might be explained due to slight differences in the assay conditions and use of the N-terminal His-Tag, which resulted in a lower K*_m_* for glucuronic acid compared with the reported data. Yoon and colleagues used a C-terminal His-Tag version of UdhAt and UdhPs. Comparing an inorganic buffer and an organic buffer system related to the kinetic parameters an increasing *K_m_* for the substrates was detected for UdhAt and UdhPs in potassium phosphate buffer. Because the other three enzymes did not show the same behaviour, an interaction between the substrate and components of the buffer is excluded. It is more likely that an interaction between phosphate ions of the buffer and the enzyme affects the binding of the substrate.

Besides the two commercially available substrates, mannuronic acid, a third substrate, was tested. Zajic (1959) reported an activity using mannuronate as substrate. Mannuronic acid was considered as an additional substrate because two of the enzymes were derived from marine microorganisms. Mannuronate is found abundantly in marine habitats as a component of polysaccharides, like alginic acid. Determination of the kinetic constants revealed no higher activity compared with GlcA and GalA. All five candidates showed an increased *K_m_* with mannuronic acid as substrate. UdhFp showed the lowest *K_m_* of all five enzymes. The *K_m_* was increased by a factor of 30 for all enzymes except for UdhOg, which only showed an increased *K_m_* by the factor 2. The assumption that the two enzymes from the marine habitat could have a better activity with mannuronic acid could not be confirmed. But contrary to the statement of Bateman and colleagues (1970), an activity of UdhPs with mannuronate as substrate could be measured.

The difference between uronate dehydrogenase of *O. granulosus* and the other four enzymes concerning *K_m_* and *k_cat_* are quite obvious. Perhaps this enzyme has different natural substrates than GlcA, GalA or ManA, which have yet to be determined. In the context of an efficient biocatalyst, UdhOg represents a poor candidate. Nevertheless, on the basis of the kinetic data and the alignment, it is possible to identify factors that are responsible. Ahn and colleagues (2012) proposed several amino acids involved in the interaction of enzyme and substrate in the active centre on the basis of the crystal structure of *C. salexigens*. Also Jörnvall and colleagues (1984) classified the glucose dehydrogenase of *Bacillus megaterium* as a member of the SDR family. Both, glucose dehydrogenase as well as uronate dehydrogenase, act on the C_1_-carbonyl function. Therefore, the low activity of all uronate dehydrogenases towards glucose or galactose is not really surprising. The carboxyl function at the C_6_ of the uronic acids appears to be the main part for interaction and stabilization of the substrate in the active centre. Within the amino acid sequence of UdhOg, two differences in the highly conserved domains were observed. The first distinctive feature appears in the β5-sheet, where an isoleucine is located instead of an asparagine, and the second at the end of the β6-sheet where a glycine is replaced by a phenylalanine. In the β5-sheet, a polar residue was replaced by an unpolar residue responsible for stabilization of the carboxyl group of the substrate. In the β6-sheet, a small residue was exchanged by a large residue, thus altering the spatial conditions. To identify the direct impact of both substitutions especially on the K*_m_* further experiments will be necessary. The different chiralities at position C2 and C4 in the three substrates result in a changed interaction within the active centre. The differences for GluA and GalA are minor, there only is one additional hydrophobic interaction for galacturonic acid possibly increasing the enzymes' affinity towards this substrate. The lowered affinity for mannuronate as substrates is derived from a steric hindrance between the C2 hydroxy function of the substrate and the carbonyl function of Ser74 (UdhAt), located between β4 and α3, resulting in a repelling force.

UdhOg also showed the most considerably differences concerning temperature stability and pH optima. It is most stable for incubation at 37°C, showing the widest temperature range, but also the most narrow pH range for optimal activity in contrast to the other four enzymes. The low temperature stability, especially of UdhPs and UdhSv is prohibitive for them to be used in cell-free biotransformation applications. Also, UdhAt represents a good biocatalyst concerning the catalytic activity; the short half-life at 37°C is a drawback. Currently, Megazyme distributes an uronate dehydrogenase in a commercial kit for standardized quantification of GlcA and GalA. The enzyme is supplied as a protein precipitate in ammonium sulphate and according to the manufacturer is stable on storage for around 2 years. No information about its stability under reaction conditions is provided. However, taken from the recommended assay conditions (application at 25°C for 10 min), a high stability is not a required feature of this enzyme. One solution to circumvent the problem of limited stability is the use of whole cells for biotransformations as shown by Mojzita and colleagues (2010). Further problems remain concerning efficient product recovery and efficient mass transfer, e.g. by active transport of substrate and the product across the cell membrane. Using isolated enzymes in combination with an efficient cofactor recycling would be a more effective way (Guterl *et al*., 2012). This would allow the use of natural low solubility of the aldaric acids as an easy purification step (Mojzita *et al*., 2010). Taking into account the properties of all enzymes, UdhFp represents the most promising candidate for a cell-free process showing a broad pH range (7–10) and high temperature stability. A biocatalytic route for the production of aldaric acids, especially glucaric acid classified as ‘top value-added chemicals’, will allow the further exploitation of renewable resources for a biobased economy. Using non food raw materials like hemicellulose and pectin will circumvent any food or fuel debate. Furthermore, the realization of cell-free processes using uronate dehydrogenase will open the avenue towards sustainable material utilization of renewable resources.

## Experimental procedures

### Reagents

Restriction enzymes, alkaline phosphatase, T4 polynucleotide kinase, Phusion® high-fidelity DNA polymerase, T4 ligase were purchased by New England Biolabs (Frankfurt, Germany). Taq polymerase was obtained from Rapidozym (Berlin, Germany). Oligonucleotides were from Thermo Scientific (Germany). DNaseI was obtained from Serva (Heidelberg, Germany). All chemicals were of analytical grade or higher quality and purchased from Sigma-Aldrich, Molekula or Carl Roth. For protein purification equipment including columns from GE Healthcare was used (Munich, Germany).

### Strains and plasmids

The following strains were used during this work; *E. coli* XL1 Blue, *E. coli* BL21(DE3), *S. viridochromogenes* DSM 40736 and *O. granulosus* DSM 15982. DNA sequences for the corresponding genes of uronate dehydrogenase (*udh*) from *A. tumefaciens* C58 (protein sequence GenBank™ DAA06454.1), *P. syringae* pv. tomato str. DC3000 (protein sequence GenBank™ ABY64888.1) and *F. pelagi* HTCC2506 (protein sequence GenBank™ ZP_01440880.1) were synthesized with optimized codon usage for expression in *E. coli* (Life Technologies, Regensburg, Germany). For cloning of the uronate dehydrogenase genes of *S. viridochromogenes* DSM 40736 (protein sequence GenBank™ ZP_07302919.1) and *O. granulosus* DSM 15982 (protein sequence GenBank™ ZP_01155501.1) genomic DNA was used as PCR template. For cloning of *udh*At the primers F-*udh*-A.t.- CAGCAAGGTCTCACAT**ATG**AAACGTCTGCTGGTTACCG and R-*udh*-A.t.- GCTCTGTTTAAAGATCGGGTTATC, for *udh*Ps the primers F-*udh*-P.s.- CAGCAAGGTCTCACAT**ATG**GCAAGCGCACATACCAC and R-*udh*-P.s.- TTTATCACCAAACGGACCGCTTGC, for *udh*Fp the primers F-*udh-*F.p.- CAGCAAGGTCTCACAT**ATG**CTGGAAAAACTGCTGATTAC and R-*udh-*F.p.- GCTATGAATCGGTTCTGCGG, for *udh*Og the primers F-*udh-*O.g.- CAGCAAGGTCTCACAT**ATG**AAGAAGATCGTCCTCACCG and R-*udh-*O.g.- CTACTTGCCCGACAGCGCCTTG and for *udh*Sv the primers F-*udh-*S.v.- CAGCAAGGTCTCACAT**ATG**CCCGCTCCCCGCACCGTTCTG and R-*udh-*S.v.- TCAGTACGGCCAGATCGGCG were used. The restriction enzyme recognition side for BsaI is underlined and the start codon is marked in bold. The reverse primers were phosphorylated using T4 polynucleotide kinase according to the supplier's manual. PCR products were digested with BsaI and cloned into pCBR, pCBRHisN and pCBRHisC, which are derivatives of pET28a (Novagen). The cloning strategy of all pET28 derivatives is described by Guterl and colleagues (2012). Ligation of the PCR products and following transformation led to the plasmids pCBR-*udh*-A.t., pCBR-*udh*-F.p., pCBR-*udh*-O.g., pCBR-*udh*-S.v., pCBRHisN-*udh*-A.t., pCBRHisN-*udh*-F.p., pCBRHisN-*udh*-O.g., pCBRHisN-*udh*-S.v., pCBRHisC-*udh*-A.t., pCBRHisC-*udh*-F.p., pCBRHisC-*udh*-O.g., pCBRHisC-*udh*-S.v. Multiplication of the plasmids was performed by *E. coli* XL1 Blue (Stratagene) in LB medium containing 30 μg ml^−1^ kanamycin. *Escherichia coli* BL21(DE3) (Novagen) was used for expression.

### Isolation of genomic DNA

The genomic DNA from *O. granulosus* DSM 15982 was isolated from cells of an overnight culture using the protocol of Chen and Kuo (1993). For the preparation of genomic DNA from *S. viridochromogenes* DSM 40736 the protocol of Saha and colleagues (2005) was used.

### Enzyme assay

The uronate dehydrogenase activity was determined photometrically by monitoring the increase of NAD(P)H at 340 nm in a Mulitskan spectrum spectrophotometer (Thermo Fisher Scientific). The reaction mixture contained 50 mM KP_i_/Tris, pH 8.0, 0.3 mM or 1 mM NAD(P)H and glucuronic or galacturonic acid as substrate. Measurement was performed at 37°C after adding the purified enzyme. One unit of enzyme activity was defined as the amount of protein that oxidizes 1 μmol of NAD(P)H/min at 37°C. Calculation of Michaelis–Menten kinetics for determination of *K*_m_ and V_max_ was done with sigma-plot 11.0 (Systat Software).

### Enzyme expression and purification

Every construct was tested for successful expression in 10 ml using LB with induction by 1 mM IPTG at an OD_600_ of 0.6 or autoinduction media under various temperatures (Studier, 2005). Optimized protein expression is exemplarily described for one enzyme and was performed for all proteins by the same procedure. *Escherichia coli* BL21(DE3) containing the plasmid of interest was grown in 200 ml of autoinduction media (Studier, 2005). The preculture was incubated in 4 ml of LB medium with 100 μg ml^−1^ kanamycin at 37°C overnight on a rotary shaker (180 r.p.m.). Expression culture was inoculated with a 1:100 dilution of overnight culture. Incubation was performed foremost 3 h at 37°C followed by incubation for 21 h at 16°C. Cells were harvested by centrifugation and resuspended in 50 mM sodium phosphate buffer (pH 8.0, 20 mM imidazole, 500 mM NaCl and 10% glycerol). Crude extracts were prepared by use of a Basic-Z Cell Disrupter (IUL Constant Systems) and subsequent addition of MgCl_2_ to a final concentration of 2.5 mM in combination with DNaseI (1 μg ml^−1^) and a following incubation for 20 min at room temperature to degrade DNA. The insoluble fraction of the lysate was removed by centrifugation (20,000 r.p.m. for 40 min at 4°C). The supernatant was filtered through a 0.45 μm syringae filter and applied to an immobilized metal ion affinity chromatography (IMAC) column, 5 ml of HisTrap™ FF, equilibrated with the resuspension buffer using the ÄKTA Purifier system. The enzyme was washed with 20 ml of resuspension buffer and eluted with 50 mM sodium phosphate buffer (pH 8.0, 500 mM imidazole, 500 mM NaCl and 10% glycerol). Aliquots of each eluted fraction were subjected to 12% SDS-Page described by Laemmli (1970). The molecular weight was calculated to be 31.21 kDa for Udh of *A. tumefaciens* (UdhAt), 32.76 kDa for Udh of *P. syringae* (UdhPs), 30.80 kDa for Udh of *F. pelagi* (UdhFp), 31.42 kDa for Udh of *S. viridochromogenes* (UdhSv) and 32.04 kDa for *O. granulosus* (UdhOg) (including the additional amino acids of a N-terminal His-Tag) using the protparam tool (Artimo *et al*. 2012). The fractions containing the eluted protein were pooled and the protein was desalted using a HiPrep™ 26/10 Desalting column, which preliminary equilibrated with 50 mM Tris-HCl pH 8.0. Protein concentrations were determined using a Bradford assay Roti®-nanoquant with BSA as standard (Carl Roth).

### Determination of kinetic parameters

To characterize and compare the five uronate dehydrogenases, enzyme activities were assayed for the oxidation of glucuronic acid, galacturonic acid and mannuronic acid at pH 8.0, 50 mM KP_i_/Tris and 37°C. For every substrate, *K*_m_ and *k*_cat_ values were determined as well as for the corresponding cofactor NAD(P)^+^. Additionally, the activity was determined at different temperatures in the range of 15–60°C using 50 mM KP_i_ pH 8.0 and 0.3 mM NAD^+^ with 5 mM glucuronic acid or 1 mM NAD^+^. In the case of UdhOg, 10 mM and 100 mM glucuronic acid were used as substrate concentration. The increase of NAD(P)H at 340 nm was determined for a time interval of 2 min using a Shimadzu UV-1800 UV-spectrophotometer (Duisburg, Germany). The influence of pH on enzyme activity was determined in the range of pH 5 to 10 with a 50 mM triple buffer consisting of equal parts of acetate/phosphate/glycine allowing the application of a single buffer system. Additional UdhFp was tested for pH-dependent enzymatic activity using an ammonium bicarbonate buffer in the range of 9.5–11.0.

### Sequence identification and selection

The public available protein sequences of the uronate dehydrogenases of *A. tumefaciens*, *P. putida* and *P. syringae* were used as query sequence for the identification of potential candidates, able to catalyse the oxidation of glucuronic and galacturonic acid. Protein sequence-based blast analysis (blastp) was performed for each enzyme (Altschul *et al*., 1997). Candidate proteins belonging to another species with a maximum identity between at least 40% and 60% were chosen.

### Structure modelling

On the basis of two available crystallized structures models, the structures for the remaining four uronate dehydrogenases were calculated using phyre2 (Kelley and Sternberg, 2009). By use of 3dligandsite the cofactor NAD^+^ was incorporated into the model structure (Wass *et al*., 2010). Comparison of the two existing and the three modelled structures was performed with pymol in combination with an alignment achieved by clustal omega (Schrodinger, 2010; Sievers *et al*., 2011). ligplot+ was used to visualize the differences in the substrate binding using the crystal structure of UdhAT (PDB: 3RFV) (Laskowski and Swindells, 2011).

### Preparation of mannuronic acid

Preparation of mannuronic acid was done as described by Spoehr (1947) using alginic acid as starting material. It is necessary to use alginic acid and not a salt preparation thereof. Starting with the gradual addition of 50 g of alginic acid to 800 ml of formic acid to prevent formation of lumps the suspension was boiled for 10 h and followed by the described purification procedure. The overall yield of d-mannuronic acid lactone was around 20% of the alginic acid used for hydrolysis. This is less than described by Spoehr, but the final crystallization procedure was only applied once. Afterwards, a solution containing mannuronic acid lactone was adjusted to pH 11 for ring opening. Directly after this, the solution was shifted to pH 8 and used for determination of kinetic parameters.

## Conflict of interest

None declared.
